# Howard Cane

**Published:** 1910-05-28

**Authors:** 


At his home, The Gables, Belvedere, Howard Cane, M.D., suddenly. Dr. Cane, a former student of University College Hospital, was physician to the Erith Cottage Hospital, and to the Belvedere Dispensary, L.C.C. Surgeon and Lecturer, and Accident Surgeon, Erith. He was formerly Resident Physician at Westminster Hospital and House Surgeon at University College Hospital. He took his diploma of M.R.C.S.Eng. in 1876, and in the following year that of M.D. Brussels and L.R.C.P. London.

